# Strength of Graphene-Coated Ni Bi-Crystals: A Molecular Dynamics Nano-Indentation Study

**DOI:** 10.3390/ma13071683

**Published:** 2020-04-04

**Authors:** Vardan Hoviki Vardanyan, Herbert M. Urbassek

**Affiliations:** Physics Department and Research Center OPTIMAS, University Kaiserslautern, Erwin-Schrödinger-Straße, D-67663 Kaiserslautern, Germany; vardanya@rhrk.uni-kl.de

**Keywords:** molecular dynamics, nickel-graphene composites, dislocation interaction with interface, interface failure

## Abstract

Nanoindentation simulations are performed for a Ni(111) bi-crystal, in which the grain boundary is coated by a graphene layer. We study both a weak and a strong interface, realized by a $30^{\circ }$ and a $60^{\circ }$ twist boundary, respectively, and compare our results for the composite also with those of an elemental Ni bi-crystal. We find hardening of the elemental Ni when a strong, i.e., low-energy, grain boundary is introduced, and softening for a weak grain boundary. For the strong grain boundary, the interface barrier strength felt by dislocations upon passing the interface is responsible for the hardening; for the weak grain boundary, confinement of the dislocations results in the weakening. For the Ni-graphene composite, we find in all cases a weakening influence that is caused by the graphene blocking the passage of dislocations and absorbing them. In addition, interface failure occurs when the indenter reaches the graphene, again weakening the composite structure.

## 1. Introduction

Graphene-metal nanocomposites are an interesting class of materials which exhibit improved mechanical properties [1]. In these, graphene—with its high in-plane elastic modulus and yield strength—is used as reinforcement component for ductile metals [2,3,4,5]. The increase in strength in the nanocomposite materials is usually attributed to the presence of interfaces that act as barriers to the propagation of dislocations [6,7,8,9].

The mechanisms underlying the interaction of dislocations with interfaces are, however, complex [10,11,12,13,14,15]. Open questions concern the transparency or opaqueness of grain boundaries to the slip of dislocations, the ability of interfaces to absorb dislocations or repel them, and in particular the issue how the interface–dislocation interaction affects the mechanical behavior of the material, and in particular its hardness. Molecular dynamics (MD) simulations have been used to unravel such mechanisms on an atomistic level. Here, besides uniaxial compression or tension tests, simulated nanoindentation offers an adequate means to introduce dislocations into the system and to study their propagation and interaction, as has been shown in previous studies of multilayered [8,16], nanolaminated [17,18], and nanotwinned [19,20,21,22] materials.

Ni constitutes a prominent metal-matrix material and has been used in a variety of both experimental and computational studies of Ni-graphene nanocomposites [23,24,25,26,27,28,29,30].

In the present work, we use MD simulation to study how the insertion of graphene sheets changes the mechanical response of poly-crystalline Ni. As a model study, we investigate here a bi-crystalline Ni system, into whose grain boundary a graphene sheet is inserted. As it is known that graphene tends to align with Ni(111) planes [24]—the lattice mismatch of graphene with the Ni(111) plane is only 2.9%—we use Ni bicrystals containing a twist grain boundary around the [111] axis. Here, three twist angles will be studied: $0^{\circ }$, corresponding to an ideal crystal, as a reference case; $30^{\circ }$, which is the grain boundary with the highest interface energy; and $60^{\circ }$, which constitutes a low-energy twin boundary. By comparing nanoindentation of such bi-crystals (Section 3) with those where the the grain boundary is filled with a graphene flake (Section 4), we can identify the mechanisms of how graphene affects the dislocation propagation in such systems.

## 2. Simulation Method

**Figure 1:**
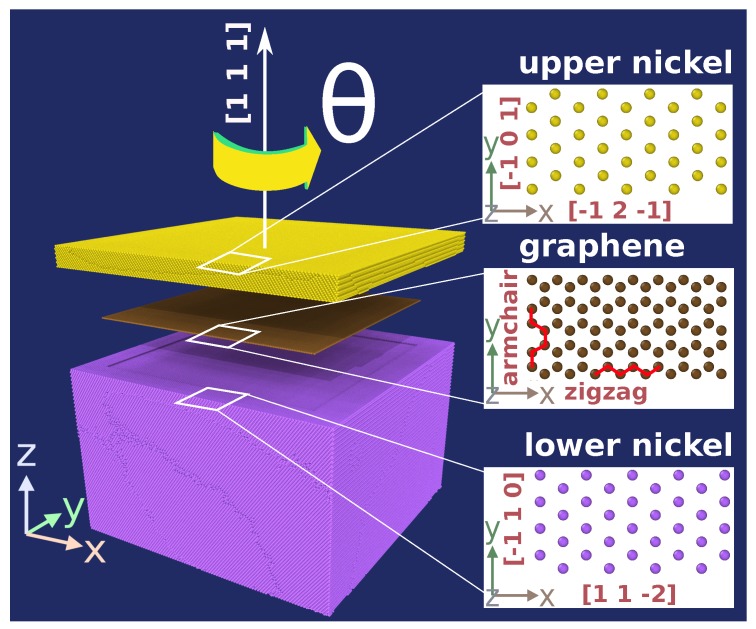
Schematic illustration of the simulation system. Both the lower Ni block (purple) and the upper Ni block (yellow) have a (111) surface; they are separated by a twist grain boundary with twist angle θ. Besides the twist angle of $60^{\circ }$, we also study $30^{\circ }$ and $0^{\circ }$, i.e., a single crystal. In the grain boundary, a graphene layer (brown) is inserted.

We describe the setup of the simulation system, see Figure 1, in three steps.

**Figure 2:**
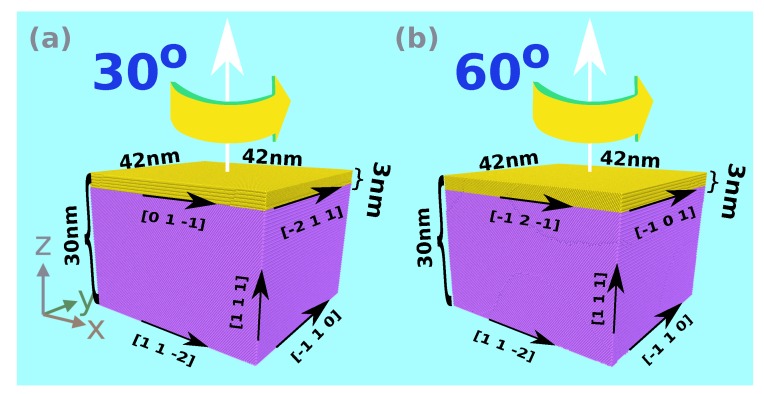
Crystallographic orientations of the grains in case of a (**a**) $30^{\circ }$ and a (**b**) $60^{\circ }$ twist boundary between the upper (yellow) and the lower (purple) Ni block. In both cases, the zigzag edge of graphene is aligned with the $\left[11\bar{2}\right]$ direction of the lower Ni block.

(i) A Ni single-crystalline block with a height of 30 nm and lateral extensions of 42 nm is constructed, containing approximately 4.9 million atoms. The surface is oriented in a [111] direction. A Cartesian system with *z* pointing in [111] direction, *x* in $\left[11\bar{2}\right]$ and *y* in $\left[\bar{1}10\right]$ direction is introduced.
(ii) A twist grain boundary is generated in this system by rotating the upper 3 nm of the Ni block by an angle θ around the [111] direction; see Figure 2. In this work, we consider $\theta =0^{\circ }$—a single crystalline system for reference—, $\theta =30^{\circ }$—a weak grain boundary with a specific energy of 0.49 Jm${}^{-2}$ [31]—and $\theta =60^{\circ }$—a strong coherent twin grain boundary with a specific energy of 0.06 Jm${}^{-2}$ [31].
  These systems will be denoted as *hm* (homointerface) systems.
(iii) A graphene flake of side length 34 nm is inserted into the grain boundary. It is aligned with the lattice of the lower Ni block, such that the zigzag and armchair edges of the graphene flake run along the $\left[11\bar{2}\right]$ and $\left[\bar{1}10\right]$ directions, respectively.
  The structures containing graphene in the grain boundary will be denoted as *g* (graphene) systems.

The created systems are relaxed by first performing energy minimization using the conjugate–gradient method. After minimization, the systems are annealed at 300 K for 50 ps. Then, the samples are cooled back to 1 mK within 50 ps. Pressure relaxation is continued for further 300 ps, until all the stress components reach values <30 MPa.

The interaction between Ni atoms is described by the embedded-atom-method interaction potential developed by Mishin et al. [32]. The carbon atoms in the graphene layer are described by the adaptive intermolecular reactive empirical bond order (AIREBO) potential of Stuart et al. [33]. Finally, the interaction between graphene layer and Ni is described by a pairwise Lennard–Jones potential described by Huang et al. [34].

Indentation simulations are performed by modeling the indenter with a repulsive potential [35] as

(1) $$V\left(r\right)=\left\{\begin{array}{ll} k{\left(R-r\right)}^{3}, & r<R\;,\\ 0, & r\geq R\;. \end{array}\right.$$

Here, *R* denotes the indenter radius, which is set to R=5 nm, and *r* is the distance of a substrate atom to the indenter. The indenter stiffness has been set to k=10 eVÅ${}^{-3}$ [35,36]. Using a displacement-controlled algorithm [37], the indenter is moved perpendicular into the surface to a final depth of 5 nm with a velocity of 20 m/s. The system is set under lateral periodic boundary conditions. The two bottommost layers of the system are fixed in order to suppress any translational movement of the Ni under indentation. The next four layers at the bottom, as well as the four outermost layers of the substrate in lateral direction, are cooled to a temperature <1 K using velocity scaling in order to minimize any thermal effects on dislocation generation or movement.

For each system, we perform five individual indentation simulations which differ from each other by the exact positioning of the indenter; it was moved randomly to another position by around ±2 Å.

The simulations are performed with the open-source Large-scale Atomic/Molecular Massively Parallel Simulator (LAMMPS) code [38] using a constant time step of 1 fs. Common-neighbor analysis (CNA) [39] is used to identify the local crystalline structure. The Open Visualization Tool (OVITO) [40] is used to visualize the simulation results.

## 3. Grain Boundaries without Graphene

In this section, we present the results of indentation into an elemental Ni bi-crystal as a reference. We consider $30^{\circ }$ and $60^{\circ }$ twist grain boundaries at a depth of 3 nm in addition to a single-crystalline Ni block.

**Figure 3:**
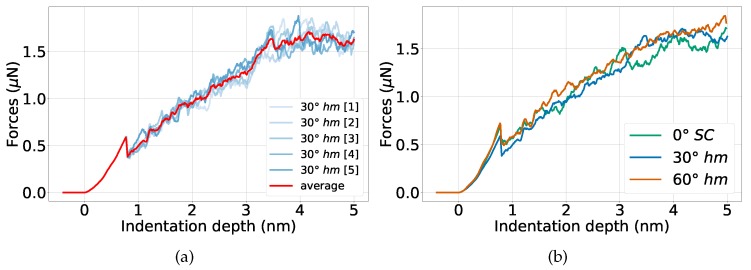
Force–depth curves for indentation into elemental Ni. (**a**) five individual simulations of indentation into a bi-crystal, containing a $30^{\circ }$ grain boundary at 3 nm depth; (**b**) comparison of the averaged force–depth curves for indentation into bi-crystals containing a $30^{\circ }$ or a $60^{\circ }$ grain boundary with that of a single crystal.

Figure 3a shows the force–depth curves for indentation into a bi-crystal containing a $30^{\circ }$ grain boundary. The results for the five individual simulations are shown as well as their average. We observe that the elastic regime as well as the initial load drop, which occurs upon dislocation nucleation, are identical in all simulations. The ensuing behavior differs somewhat among the individual simulations as it is caused by the individual events of dislocation nucleation, migration and interaction. The spread of the curves around the average increases with indentation depth and reaches values of around ±0.1 μN at the deepest indentation, 5 nm. Note that the force saturates towards the deepest indentation since then we have indented up to the equator of the spherical tip. In the following, we will base our analysis on the averaged data.

Since, in all our studies, we use the same indenter and target material, we can use the force–depth curves to identify the material response. In particular, the momentary contact pressure—given by the quotient of the force and the cross-sectional area of the indenter at a specified depth—contains no further information than the force–depth curves. Hence, we can interpret an increase of the force at specified depth as an apparent hardening of the material. In addition, deviations at the depth of the first load drop are caused by the incipient plasticity, i.e., the nucleation of the first dislocations in the material.

In Figure 3b, we compare the force–depth curves for indenting into bi-crystals containing a $30^{\circ }$ or a $60^{\circ }$ twist grain boundary with that of a single crystal.

**Figure 4:**
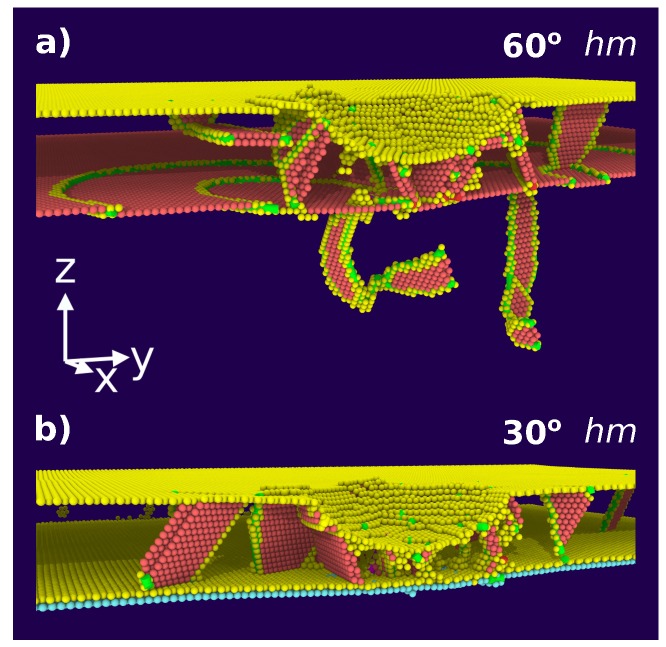
Dislocation network building up after indentation to a depth of 2.1 nm into a Ni bi-crystal containing a (**a**) $60^{\circ }$ and (**b**) $30^{\circ }$ grain boundary at 3 nm depth. Atoms are colored according to common-neighbor analysis. Red: stacking faults; yellow (cyan): defective atoms in the upper (lower) Ni grain. Fcc atoms have been removed for clarity. Green lines shows Shockley partials.

Let us first discuss the behavior of the $60^{\circ }$ twist grain boundary. This is a coherent Σ3 twin boundary with an extremely small specific energy of only 0.06 Jm${}^{-2}$ [31]. It therefore constitutes an example of a strong grain boundary. In fact, the initial load drop as well as the ensuing evolution of the force with depth—until the indenter reaches the grain boundary at 3 nm—are similar to the single-crystalline case. As Figure 4a shows, the dislocations nucleated in the upper Ni grain can easily propagate into the lower grain as the interface coherency allows dislocation slip; the first dislocation transmission occurs already at 1.4 nm. However, once the indenter has passed through the grain boundary, the indentation force appears to be systematically higher than for the single-crystalline case. This is due to the fact that now new dislocation nucleation in the lower Ni grain starts.

The hardening that we observe for the $60^{\circ }$ twist grain boundary is caused by the interaction of dislocations with the twin boundary. Indeed, as Figure 4a shows, the twin boundary is transparent and dislocations can be transmitted into the lower grain. These results are in agreement with previous studies [41,42,43,44]. However, as Jin et al. [41,42] report for Cu and Ni, the dislocation transmission through the twin boundary requires external strain. Although their study models a simpler scenario where screw and non-screw dislocations interact with the twin boundary, their argumentation can be extended for the case where dislocations interact with twin boundary during nanoindentation. Thus, Kulkarni et al. [44] observed force hardening in the case of nanoindentation into Cu containing a twin boundary. We conclude that the hardening we observe by the introduction of a twin boundary into single-crystalline Ni is caused by the interface barrier strength felt by dislocations upon gliding through the twin boundary; see also the review by Wang et al. [17].

The indentation into the upper grain moves the grain boundary towards the lower grain. This is seen in Figure 4a by monatomic steps appearing in the grain boundary, which have been denoted as twinning partial slips [22,45]; these references argue that this grain-boundary mechanism weakens the material response as it makes the material yield easier. In our case, this process is not dominant, as we observe an overall hardening caused by the introduction of a $60^{\circ }$ twist grain boundary into Ni.

The $30^{\circ }$ twist grain boundary shows a quite different behavior. Already at the first load drop, it features a smaller strength, see Figure 3b. Such a behavior was already seen in previous simulations [46,47]. It is caused by the fact that the grain boundary acts as a defect that lowers the force needed to induce dislocations. The smaller strength of the $30^{\circ }$ boundary persists up to indentation depths of 3 nm. The corresponding dislocation network is shown in Figure 4b; dislocations are only created in the upper grain as the interface is opaque to dislocation passage. Dislocations are absorbed in the grain boundary resulting in a weakening behavior.

**Figure 5:**
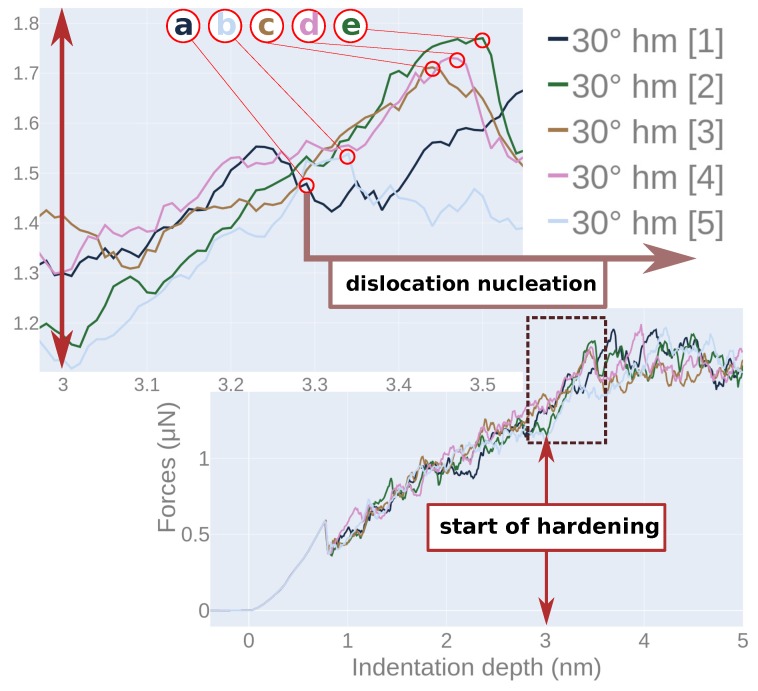
Force–depth curves for indentation into Ni containing a $30^{\circ }$ *hm* grain boundary in each individual indentation event, cf. Figure 3a. Upper insert shows a zoom into the indentation region where hardening starts. Circled points in the zoom, **a–e**, indicate indentation depth where dislocations start nucleating in the lower Ni block.

**Figure 6:**
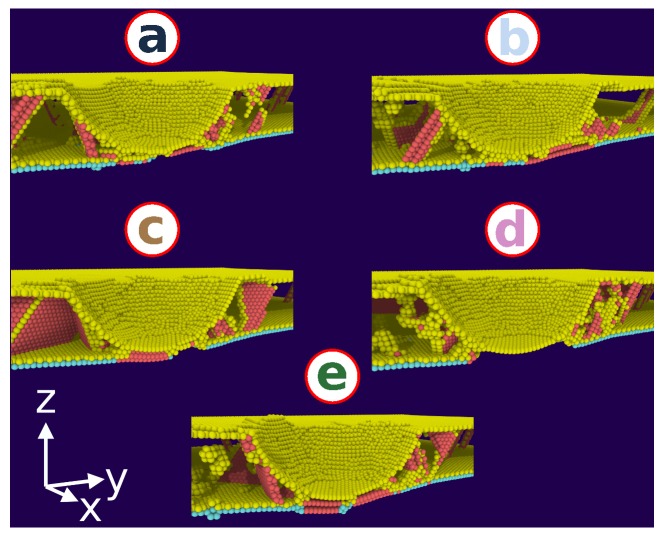
Snapshots showing the microstructure induced in Ni containing a $30^{\circ }$ *hm* grain boundary at the indentation points, **a–e**, of Figure 5. Colors denote lattice defects as in Figure 4.

After the indenter passed the interface, at indentation depths >3 nm, the force rises until it reaches the level of the single-crystalline sample. While at 3 nm indentation depth, the lower grain was still dislocation-free (see Figure 4b), further penetration of the indenter into the grain boundary produces dislocations in the bottom grain with the typical accompanying force drop. We show this at the point immediately preceding dislocation nucleation in the lower grain for the individual simulations in Figure 5—these points are marked by a–e—and illustrate the changes in the local lattice structure in Figure 6. Indeed, the force is lowered after the marked points. In addition, we observe that the local lattice structure at the interface changes at these points; in some cases—points a, b, c, e—stacking faults develop, while at point d the interface even changes to a perfect fcc structure, i.e., it disappears locally. This transformation under the influence of the indenter results in hardening.

We conclude that the indentation behavior of a Ni bi-crystal containing a strong (coherent) grain boundary is similar to the single crystal, since the grain boundary is transparent to dislocation slip. A slight hardening is induced by the interface barrier strength felt by dislocations upon gliding through the twin boundary. On the other hand, a Ni bi-crystal containing a weak (opaque) grain boundary, on the other hand, induces a weakening in that dislocation absorption at the interface lets the force drop. Only after the indenter passed the interface, the force increases again to the level of the single-crystalline values.

## 4. Grain Boundary Filled with Graphene

In previous work [30], we studied the effect of a graphene sheet in a Ni(111) crystal, corresponding to a $0^{\circ }$ twist boundary in the notation of the present work. We found that the graphene sheet leads to a weakening of the indentation force; only when the indenter touches the flake, strong hardening is observed by the increase of the force. The reason for the weakening was argued to be due to an attractive interaction of the graphene with the Ni dislocations; when the dislocations arrive at the Ni-graphene interface, they induce height depressions of the graphene that alleviate the pressure in the top Ni layer and weaken the force on the indenter.

**Figure 7:**
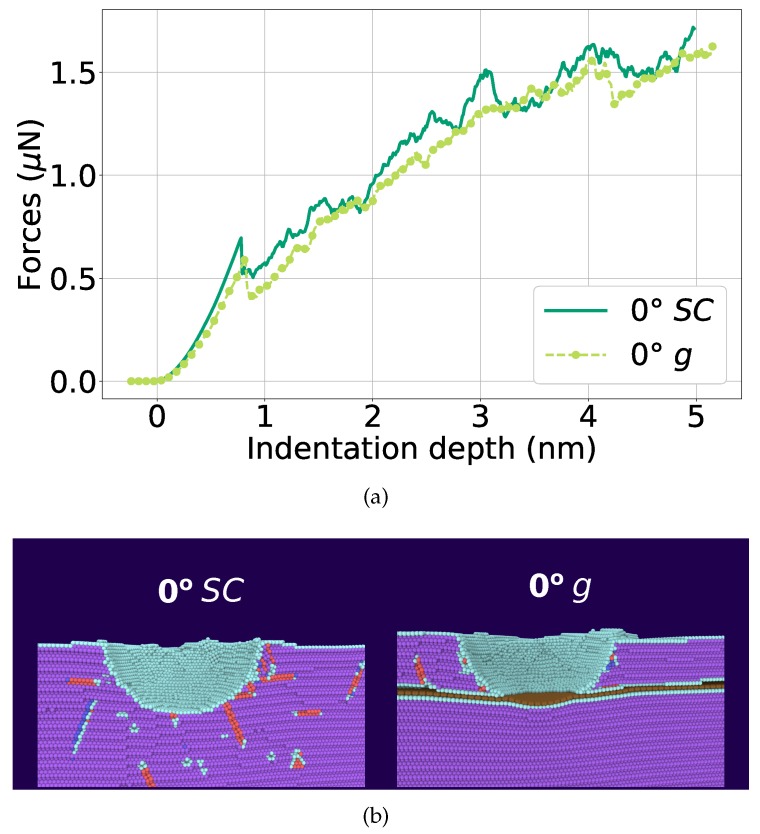
Comparison of the indentation into single-crystalline Ni (SC) with Ni containing graphene *g* at a depth of 3 nm. (**a**) averaged force–depth curves; (**b**) snapshots showing the microstructure at an indentation depth of d=4.1 nm, immediately before dislocation nucleation in the lower Ni block. Atoms are colored according to common-neighbor analysis. Purple: fcc; red: stacking faults; cyan: other defects; brown: graphene.

Figure 7a takes up this case again, now with improved statistics (averaged over five simulations). It is observed that, basically throughout the entire range of indentation depths, insertion of graphene reduces the force required for indentation. This already occurs at the point of initial load drop (at 8 Å) and persists also after the indenter touched the graphene. This is due to the fact that the graphene acts as a defect that lowers the force needed to induce dislocations. Figure 7b compares dislocation emission for an indentation depth of 4.1 nm for the graphene-loaded and the elemental Ni case; clearly the graphene layer blocks the transmission of dislocations and thus reduces the force necessary for indentation.

**Figure 8:**
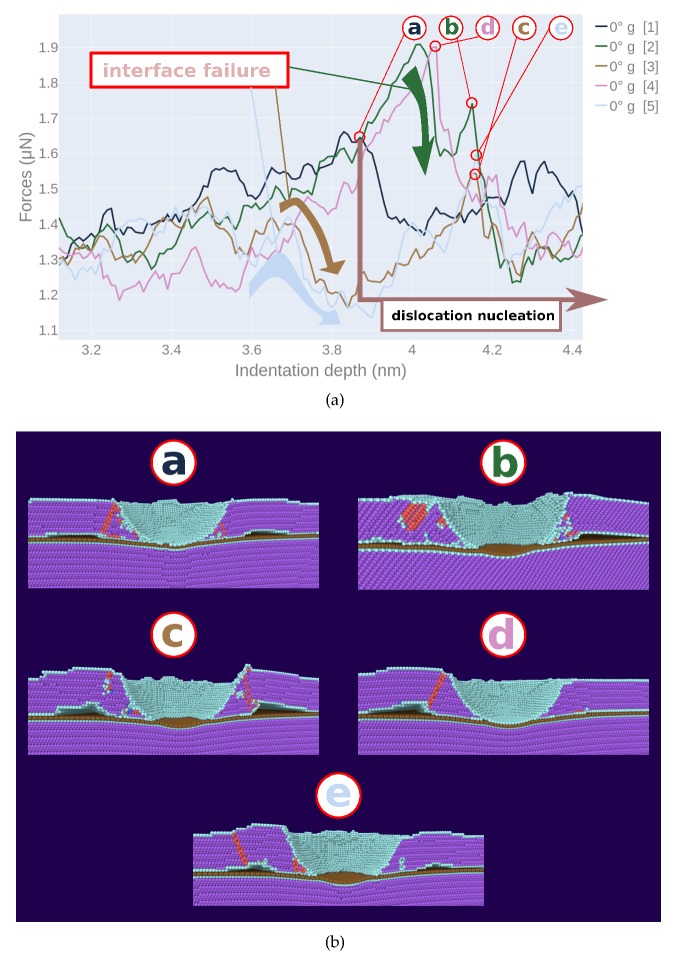
Indentation into single-crystalline Ni containing graphene ($0^{\circ }$ *g*) at a depth of 3 nm. (**a**) force–depth curves for each individual indentation event. Circled points, **a–e**, indicate the indentation depth where dislocations start nucleating in the lower Ni block. (**b**) snapshots showing the microstructure induced at the indentation points, **a–e**, in panel (**a**). Atoms are colored as in Figure 7.

Figure 7a showed only the average data. We display in Figure 8a individual force–depth curves for indentation depths beyond 3 nm, i.e., when the indenter touched the graphene. Pertinent snapshots are provided in Figure 8b at the points where dislocations start nucleating in the lower Ni block. Wide variations between the individual indentations are visible. In all cases, interface failure occurs when the graphene sheet bows so strongly that Ni loses contact with it. Note that this occurs at a maximum of the force, since, immediately after these points, dislocations nucleate in the lower Ni grain reducing the strain and the indentation force.

**Figure 9:**
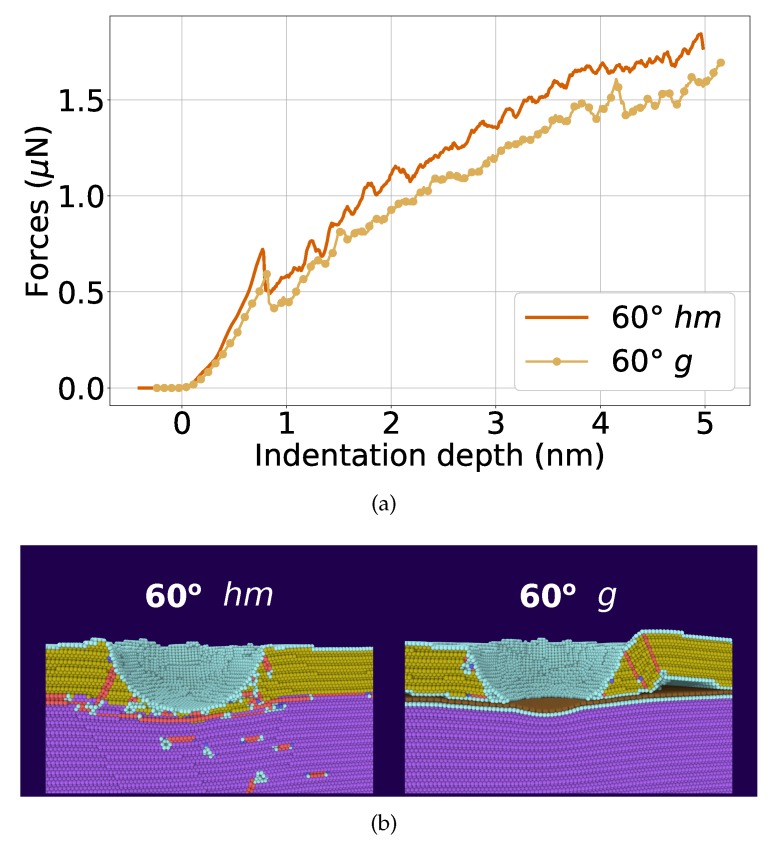
Comparison of the indentation into a Ni bi-crystal with a $60^{\circ }$ twist boundary without *hm* and with *g* graphene. (**a**) averaged force–depth curves; (**b**) snapshots showing the microstructure at an indentation depth of d=4.2 nm. Atoms are colored according to common-neighbor analysis. Purple: fcc (lower Ni block); gold: fcc (lower Ni block); red: stacking faults; cyan: other defects; brown: graphene.

The case of a $60^{\circ }$ twist boundary with and without graphene is analyzed in Figure 9, where the averaged force–indentation data are shown in Figure 9a. Here, the decrease of the indentation force upon insertion of graphene into the grain boundary is seen even more strongly than in Figure 7a. A view of the dislocations generated is shown in Figure 9b; clearly dislocations can penetrate the twin boundary in elemental Ni, but not the graphene sheet. This demonstrates that again the blocking of dislocations by the graphene sheet is responsible for the reduced force necessary for indentation of the graphene-Ni composite.

These two examples of strong grain boundaries show that insertion of graphene reduces their mechanical qualities. The main mechanism is blocking of dislocation slip through the interface.

**Figure 10:**
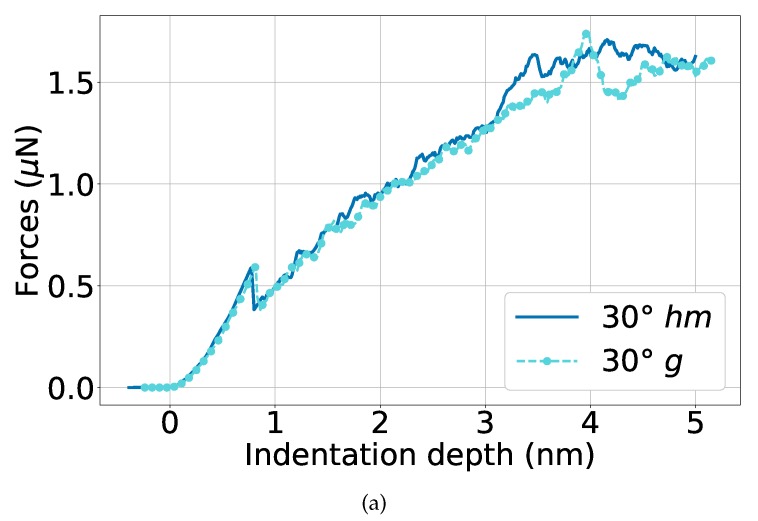

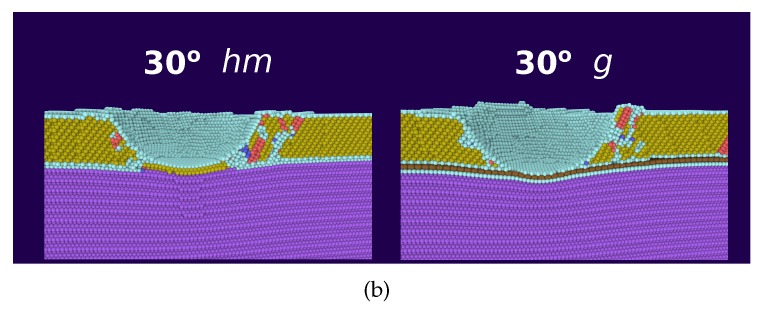
Comparison of the indentation into a Ni bi-crystal with a $30^{{}^{\circ}}$ twist boundary without *hm* and with *g* graphene. (**a**) averaged force–depth curves; (**b**) snapshots showing the microstructure immediately before dislocation nucleation in the lower Ni block: at d=3.4 nm (*hm*) and at *d* = 4.0 nm (*g*). Atoms are colored as in Figure 9.

Finally, the case of a weaker interface, the $30^{\circ }$ twist boundary, is discussed in Figure 10a. Here, astonishingly small differences between the case with and without graphene show up; note that in particular the peak at initial load drop is identical in both cases. The reason hereto is that the $30^{\circ }$ twist boundary in elemental Ni already blocks dislocation slip, and the insertion of graphene cannot improve this situation. This scenario is displayed in the snapshots in Figure 10b, where the indentation depths were chosen just immediately before dislocation nucleation in the lower Ni grain.

We conclude that the action of graphene in weak grain boundaries is minor, since dislocation slip is not possible throughout such boundaries anyway.

## 5. Conclusions

We studied nanoindentation into a Ni bi-crystal and compared to the case where the grain boundary was filled with a graphene sheet. We obtained the following findings:

1. Individual indentation events may strongly differ from each other depending on the exact indentation point. We show that it is necessary to average over sufficiently many indentation events and to discuss the average data.
2. Taking single-crystalline Ni as a reference, low-energy—i.e., strong—twin boundaries may even have increased strength. This is caused since—while these boundaries are transparent to dislocation slip—a slight hardening is induced by the interface barrier strength felt by dislocations upon gliding through the twin boundary.
3. Higher-energy, i.e., weaker, interfaces that are opaque to dislocation slip require smaller indentation forces as they confine the indention-produced dislocations.
4. The insertion of graphene cannot improve the quality of single-crystalline graphene or of low-energy twin boundaries; the indentation force rather decreases. This is caused because graphene now blocks dislocation slip.
5. Graphene insertion into a weak boundary does only negligibly change the force needed to indent.
6. Once the indenter touches the graphene flake, it strongly bows out and can detach from the Ni matrix. This interface failure again reduces the force acting on the indenter.

## References

[1] Guo Q., Kondoh K., Han S.M. (2019). Nanocarbon-reinforced metal-matrix composites for structural applications. MRS Bull..

[2] Ramanathan T., Abdala A.A., Stankovich S., Dikin D.A., Herrera-Alonso M., Piner R.D., Adamson D.H., Schniepp H.C., Chen X., Ruoff R.S. (2008). Functionalized graphene sheets for polymer nanocomposites. Nat. Nanotechnol..

[3] Zhang P., Ma L., Fan F., Zeng Z., Peng C., Loya P.E., Liu Z., Gong Y., Zhang J., Zhang X. (2014). Fracture toughness of graphene. Nat. Commun..

[4] Xiong D.B., Cao M., Guo Q., Tan Z., Fan G., Li Z., Zhang D. (2015). Graphene-and-copper artificial nacre fabricated by a preform impregnation process: Bioinspired strategy for strengthening-toughening of metal matrix composite. ACS Nano.

[5] Yang Z., Wang D., Lu Z., Hu W. (2016). Atomistic simulation on the plastic deformation and fracture of bio-inspired graphene/Ni nanocomposites. Appl. Phys. Lett..

[6] Misra A., Hirth J.P., Hoagland R.G. (2005). Length-scale-dependent deformation mechanisms in incoherent metallic multilayered composites. Acta Mater..

[7] Liu X., Wang F., Wang W., Wu H. (2016). Interfacial strengthening and self-healing effect in graphene-copper nanolayered composites under shear deformation. Carbon.

[8] Feng Q., Song X., Xie H., Wang H., Liu X., Yin F. (2017). Deformation and plastic coordination in WC-Co composite–Molecular dynamics simulation of nanoindentation. Mater. Des..

[9] Shuang F., Aifantis K.E. (2020). Relating the strength of graphene/metal composites to the graphene orientation and position. Scr. Mater..

[10] Lu K., Lu L., Suresh S. (2009). Strengthening Materials by Engineering Coherent Internal Boundaries at the Nanoscale. Science.

[11] Wu Z.X., Zhang Y.W., Srolovitz D.J. (2009). Dislocation-twin interaction mechanisms for ultrahigh strength and ductility in nanotwinned metals. Acta Mater..

[12] Wang J., Misra A. (2011). An overview of interface-dominated deformation mechanisms in metallic multilayers. Curr. Opin. Solid State Mater. Sci..

[13] Li N., Wang J., Misra A., Zhang X., Huang J.Y., Hirth J.P. (2011). Twinning dislocation multiplication at a coherent twin boundary. Acta Mater..

[14] Beyerlein I.J., Demkowicz M.J., Misra A., Uberuaga B.P. (2015). Defect-interface interactions. Prog. Mater. Sci..

[15] Weng S., Ning H., Hu N., Yan C., Fu T., Peng X., Fu S., Zhang J., Xu C., Sun D. (2016). Strengthening effects of twin interface in Cu/Ni multilayer thin films—A molecular dynamics study. Mater. Des..

[16] Zhao Y., Peng X., Fu T., Sun R., Feng C., Wang Z. (2015). MD simulation of nanoindentation on (001) and (111) surfaces of Ag-Ni multilayers. Phys. E.

[17] Wang J., Zhou Q., Shao S., Misra A. (2017). Strength and plasticity of nanolaminated materials. Mater. Res. Lett..

[18] Li N., Wang J., Misra A., Huang J.Y. (2012). Direct Observations of Confined Layer Slip in Cu/Nb Multilayers. Microsc. Microanal..

[19] Lu L., Chen X., Huang X., Lu K. (2009). Revealing the Maximum Strength in Nanotwinned Copper. Science.

[20] Li X., Wei Y., Lu L., Lu K., Gao H. (2010). Dislocation nucleation governed softening and maximum strength in nano-twinned metals. Nature.

[21] You Z., Li X., Gui L., Lu Q., Zhu T., Gao H., Lu L. (2013). Plastic anisotropy and associated deformation mechanisms in nanotwinned metals. Acta Mater..

[22] Fu T., Peng X., Chen X., Weng S., Hu N., Li Q., Wang Z. (2016). Molecular dynamics simulation of nanoindentation on Cu/Ni nanotwinned multilayer films using a spherical indenter. Sci. Rep..

[23] Chang S.W., Nair A.K., Buehler M.J. (2013). Nanoindentation study of size effects in nickel-graphene nanocomposites. Philos. Mag. Lett..

[24] Kuang D., Xu L., Liu L., Hu W., Wu Y. (2013). Graphene-nickel composites. Appl. Surface Sci..

[25] Kim Y., Lee J., Yeom M.S., Shin J.W., Kim H., Cui Y., Kysar J.W., Hone J., Jung Y., Jeon S. (2013). Strengthening effect of single-atomic-layer graphene in metal-graphene nanolayered composites. Nat. Commun..

[26] Muller S.E., Nair A.K. (2016). Dislocation Nucleation in Nickel-Graphene Nanocomposites Under Mode I Loading. JOM.

[27] Yazdandoost F., Yari Boroujeni A., Mirzaeifar R. (2017). Nanocrystalline nickel-graphene nanoplatelets composite: Superior mechanical properties and mechanics of properties enhancement at the atomistic level. Phys. Rev. Mater..

[28] Muller S.E., Santhapuram R.R., Nair A.K. (2018). Failure mechanisms in pre-cracked Ni-graphene nanocomposites. Comput. Mater. Sci..

[29] Weng S., Ning H., Fu T., Hu N., Zhao Y., Huang C., Peng X. (2018). Molecular dynamics study of strengthening mechanism of nanolaminated graphene/Cu composites under compression. Sci. Rep..

[30] Vardanyan V.H., Urbassek H.M. (2019). Dislocation interactions during nanoindentation of nickel-graphene nanocomposites. Comput. Mater. Sci..

[31] Olmsted D.L., Foiles S.M., Holm E.A. (2009). Survey of computed grain boundary properties in face-centered cubic metals: I. Grain boundary energy. Acta Mater..

[32] Mishin Y., Farkas D., Mehl M.J., Papaconstantopoulos D.A. (1999). Interatomic potentials for monoatomic metals from experimental data and ab initio calculations. Phys. Rev. B.

[33] Stuart S.J., Tutein A.B., Harrison J.A. (2000). A reactive potential for hydrocarbons with intermolecular interactions. J. Chem. Phys..

[34] Huang S.P., Mainardi D.S., Balbuena P.B. (2003). Structure and dynamics of graphite-supported bimetallic nanoclusters. Surface Sci..

[35] Kelchner C.L., Plimpton S.J., Hamilton J.C. (1998). Dislocation nucleation and defect structure during surface indentation. Phys. Rev. B.

[36] Ziegenhain G., Hartmaier A., Urbassek H.M. (2009). Pair vs many-body potentials: Influence on elastic and plastic behavior in nanoindentation of fcc metals. J. Mech. Phys. Sol..

[37] Ruestes C.J., Bringa E.M., Gao Y., Urbassek H.M., Tiwari A., Natarajan S. (2017). Molecular dynamics modeling of nanoindentation. Applied Nanoindentation in Advanced Materials.

[38] Plimpton S. (1995). Fast Parallel Algorithms for Short-Range Molecular Dynamics. J. Comput. Phys..

[39] Stukowski A. (2012). Structure identification methods for atomistic simulations of crystalline materials. Model. Simul. Mater. Sci. Eng..

[40] Stukowski A. (2010). Visualization and analysis of atomistic simulation data with OVITO—The Open Visualization Tool. Model. Simul. Mater. Sci. Eng..

[41] Jin Z.H., Gumbsch P., Ma E., Albe K., Lu K., Hahn H., Gleiter H. (2006). The interaction mechanism of screw dislocations with coherent twin boundaries in different face-centred cubic metals. Scr. Mater..

[42] Jin Z.H., Gumbsch P., Albe K., Ma E., Lu K., Gleiter H., Hahn H. (2008). Interactions between non-screw lattice dislocations and coherent twin boundaries in face-centered cubic metals. Acta Mater..

[43] Kulkarni Y., Asaro R.J. (2009). Are some nanotwinned fcc metals optimal for strength, ductility and grain stability?. Acta Mater..

[44] Kulkarni Y., Asaro R.J., Farkas D. (2009). Are nanotwinned structures in fcc metals optimal for strength, ductility and grain stability?. Scr. Mater..

[45] Liu Q., Deng L., Wang X. (2016). Interactions between prismatic dislocation loop and coherent twin boundary under nanoindentation investigated by molecular dynamics. Mater. Sci. Eng. A.

[46] Tsuru T., Kaji Y., Matsunaka D., Shibutani Y. (2010). Incipient plasticity of twin and stable/unstable grain boundaries during nanoindentation in copper. Phys. Rev. B.

[47] Voyiadjis G.Z., Yaghoobi M. (2016). Role of grain boundary on the sources of size effects. Comput. Mater. Sci..

